# Editorial: Systems biology, women in science 2021/22: translational systems biology and *in silico* trials

**DOI:** 10.3389/fsysb.2023.1293298

**Published:** 2023-09-29

**Authors:** Jane A. Leopold, Madhavi K. Ganapathiraju, Naveena Yanamala

**Affiliations:** ^1^ Brigham and Women’s Hospital, Harvard Medical School, Boston, MA, United States; ^2^ Department of Biomedical Informatics, University of Pittsburgh, Pittsburgh, PA, United States; ^3^ Robert Wood Johnson Medical School, Rutgers University, New Brunswick, NJ, United States

**Keywords:** systems biology, translational systems biology, women, *in silico* models, omics

It is increasingly recognized that there are relatively few women in science, technology, engineering, and mathematics (STEM) fields, and those who do choose to pursue careers in STEM face substantial hurdles throughout their academic careers. Women are less likely than men to be credited for their scientific research and often suffer from the “Matilda Effect,” a pervasive bias that attributes the work of women to their male colleagues (Rossiter, 1993). Women are also less likely to be named as co-authors on scientific manuscripts in all STEM fields at any stage of their careers. This problem is compounded by the fact that the percentage of women who are journal editors is low and has been estimated at less than 15% of all scientific journals (Liu F et al., 2023).

To address some of these limitations, a Research Topic in the field of systems biology was commissioned to highlight some of the cutting-edge research being done by women in the areas of translational systems biology and silico trials. The Research Topic was edited by three women and includes four original articles authored by 21 individuals. A total of eight women, ranging from students to senior academicians, served as the first and senior authors.

In the first article on the Research Topic, “*Systems biology of asphalt pollutants and their molecular targets*,” Rozewski et al. examined the effect of asphalt on human health and disease. Asphalt, which is an aggregate that contains thousands of different compounds, is widely used to pave roads, roof houses, or weatherproof structures and constitutes an environmental exposure for a large swath of the population. As asphalt ages, volatile organic compounds are released; these can have potentially deleterious effects on human health. In the article, the consequences of exposure to asphalt emissions and toxicity studies following dermal or inhalation exposure are reviewed, and supporting evidence is provided to show that asphalt exposure can lead to dermal or airway irritation and changes in gene expression. The investigators modeled the relationship between asphalt emissions, health effects, and biomolecular targets using network analysis. This network illustrates how some components of asphalt are linked to changes in gene expression that, in turn, have been linked to cardiovascular diseases such as myocardial infarction, peripheral arterial disease, or hypertension, and malignancies such as cervical, lung, or prostate cancer. Based on these previously unrecognized relationships, the authors call for further studies of the selected volatile compounds released from aging asphalt that are toxic to understand their health effects in order to modify these emissions.

Vardhan et al. published “*Evaluation of intracoronary hemodynamics identifies perturbations in vorticity*,” a study that used computational fluid dynamics to model coronary artery hemodynamics and identify hemodynamic features that differ between coronary arteries with and without stenoses. In this study, coronary angiograms from patients undergoing cardiac catheterization and pressure gradient measurement across coronary artery stenosis (a clinical test known as fractional flow reserve testing that is used to decide whether a therapeutic intervention is needed) were used to create 3-dimensional geometries for *in silico* analysis. Intracoronary hemodynamics were analyzed using the massively parallel fluid solver HARVEY, and simulation results were validated using particle image velocimetry and clinical measurements obtained during cardiac catheterization. These analyses identified significant differences in velocity, shear stress, and vorticity in a coronary artery with a stenosis compared to one without a stenosis from the same individual. The simulations also revealed that there were differences in vorticity in a normal-appearing coronary artery from patients who had stenosis in another vessel compared to normal coronary arteries from a control subject with no coronary artery disease. This study demonstrates the added value of *in silico* studies using computational fluid dynamics to analyze intracoronary hemodynamic profiling based on medical imaging data.

In “*Investigating the comorbidity of COPD and tuberculosis, a computational study*,” Sershen et al. used a multi-scale model and *in silico* simulations to investigate the mechanisms by which individuals with chronic obstructive pulmonary disease (COPD) are at greater risk for harboring a tuberculosis infection. The researchers used a validated, physiologically relevant, agent-based model of *Mycobacterium tuberculosis* (Mtb) infection and incorporated inflammatory cell recruitment, pulmonary dynamics, and variations in cytokines and chemokines into the model to evaluate Mtb clearance or retention among patients with COPD and lung damage with or without combustible tobacco use. A total of 63 COPD hosts with reduced lung capacity and varying degrees of emphysema were studied via simulations. The modeling demonstrated that in the presence of COPD, there was a greater likelihood of disseminated Mtb infection and retention of Mtb, as opposed to the clearance of infection that was typical of the non-COPD models. The study also showed that there was no real compounding effect of smoking on the immune response to Mtb. Thus, *in silico* modeling can predict both the biology and the outcome of Mtb infection in hosts with different degrees of lung function.

Chahine and Le Roch complete the series with a review of platforms and technologies for omics analyses of the malaria parasite, *Plasmodium falciparum*, and describe systems-based methodologies used for discovery in an article entitled “*Decrypting the complexity of the human malaria parasite biology through systems biology approaches*.” The review details the *Plasmodium* life cycle, the history of the sequencing of the *Plasmodium* genome, and the *Plasmodium* transcriptome, proteome, and metabolome. The review also includes a description of tools and technologies for DNA sequencing, chromatin interactions and assembly, chromosomal architecture, and RNA profiling and sequencing platforms. The authors then provide a comprehensive workflow of how omics profiling, data analysis, bioinformatics, and mathematical modeling can be used to understand host-parasite interactions and identify relevant targets for clinical diagnostics and drug therapies, and for the development of anti-malarial therapies and vaccines.

Taken together, these studies exemplify systems biology research focused on *in silico* trials that were conducted by talented female investigators in STEM. This Research Topic has been viewed several thousand times and is a gateway to a field that will benefit from the continued contributions of female academicians. We are proud to highlight the outstanding research accomplishments of these women in STEM and look forward to more female colleagues contributing to the growing field of systems biology.
